# Comparison between brain CT and MRI for voxel-based morphometry of Alzheimer's disease

**DOI:** 10.1002/brb3.146

**Published:** 2013-06-30

**Authors:** Etsuko Imabayashi, Hiroshi Matsuda, Takeshi Tabira, Kunimasa Arima, Nobuo Araki, Kenji Ishii, Fumio Yamashita, Takeshi Iwatsubo, Japanese Alzheimer's Disease Neuroimaging Initiative

**Affiliations:** 1 Department of Radiology, Tokyo Metropolitan Geriatric Hospital and Institute of Gerontology35-2 Sakaecho, Itabashi-ku, Tokyo, Japan; 2 Department of Nuclear Medicine, Saitama Medical University International Medical CenterSaitama, Japan; 3 Integrative Brain Imaging Center, National Center of Neurology and PsychiatryTokyo, Japan; 4 Department of Diagnosis, Prevention and Treatment of Dementia, Graduate School of Medicine, Juntendo UniversityTokyo, Japan; 5 Department of Psychiatry, National Center Hospital, National Center of Neurology and PsychiatryTokyo, Japan; 6 Department of Neurology, Saitama Medical University HospitalSaitama, Japan; 7 Department of Positron Medical Center, Tokyo Metropolitan Institute of GerontologyTokyo, Japan; 8 Division of Ultrahigh Field MRI, Core of Multidisciplinary Research for Medical Imaging, Institute for Biomedical Sciences of Iwate Medical UniversityIwate, Japan; 9 Department of Neuropathology and Neuroscience, Graduate School of Pharmaceutical Sciences, University of TokyoTokyo, Japan

**Keywords:** Alzheimer's disease, CT, PIB, VBM

## Abstract

The voxel-based morphometry (VBM) technique using brain magnetic resonance imaging (MRI) objectively maps gray matter loss on a voxel-by-voxel basis after anatomic standardization. In patients with Alzheimer's disease (AD), reductions of gray matter volume, mainly in the medial temporal structures, have been reported; however, inhomogeneity and geometric distortion of the field intensity hampers the reproducibility of MRI. In the present study, we developed a novel computed tomography (CT)-based VBM method and used this technique to detect volume loss in AD patients as compared with normal controls. The results were compared with MRI-based VBM using the same subjects. Pittsburgh Compound B (^11^C-PIB) positron emission tomography (PET)/CT was performed and two experts in neuro-nuclear medicine judged whether regional amyloid β load was consistent with a diagnosis of AD. Before the injection of ^11^C-PIB, high-quality CT scans were obtained using the same PET/CT equipment. MRI was performed within a mean interval of 25.1 ± 8.2 days before the PET/CT scan. Using statistical parametric mapping 8 (SPM8), the extracted gray matter images from CT and MRI were spatially normalized using a gray matter template and smoothed using a Gaussian kernel. Group comparisons were performed using SPM8 between five ^11^C-PIB-positive patients with probable AD and seven ^11^C-PIB-negative age-matched controls with normal cognition. Gray matter volumes in the bilateral medial temporal areas were reduced in the AD group as compared with the cognitively normal group in both CT-based VBM (in the left; *P* < 0.0001, cluster size 2776 and in the right; *P* < 0.0001, cluster size 630) and MRI-based VBM (in the left; *P* < 0.0001, cluster size 381 and in the right, *P* < 0.0001, cluster size 421). This newly developed CT-based VBM technique can detect significant atrophy in the entorhinal cortex in probable AD patients as previously reported using MRI-based VBM. However, CT-VBM was more sensitive and revealed larger areas of significant atrophy than MR-VBM.

## Introduction

Macroscopically, the brain has a simple structure, despite its complex functions. Morphologically, brain structures consist mainly of gyri and sulci, with these structures being quite common in human beings. This simplicity makes it easier to normalize the brain to an anatomically standardized space and to introduce voxel-based statistical analysis. Histologically, the majority of brain tissue consists of gray matter, white matter, and cerebrospinal fluid space. Gray matter thickness reflects the number of residual neurons. Commonly, brain magnetic resonance imaging (MRI) has been used to derive anatomical and tissue volume information, especially in gray and white matter.

The voxel-based morphometry (VBM) technique objectively maps gray matter loss on a voxel-by-voxel basis after anatomic standardization. This is one of the simplest methods available to avoid subjectivity and dependence on an a priori hypothesis and to adopt the principle of data-driven analysis (Ashburner and Friston 2000). In patients with Alzheimer's disease (AD), a significant reduction of gray matter volume in the hippocampal formation and entorhinal cortex has been described (Ohnishi et al. 2001; Hirata et al. 2005). For the voxel-wise comparison of gray matter between two groups of subjects, MRI has been used as a matter of course.

In the present study, we developed a novel computed tomography (CT)-based VBM (CT-VBM) technique. Brain CT has more homogeneity and much less distortion than MRI, even when using different machines or scan protocols. It is also relatively economical and widely available. Moreover, nowadays, CT data are easily available from a routine positron emission tomography (PET)/CT study. In the present study, we also compared the results from CT-VBM with those from MRI-based VBM (MR-VBM) using the same individuals.

## Materials and Methods

### Subjects

All of the subjects were enrolled in the Japanese Alzheimer's Disease Neuroimaging Initiative (J-ADNI). The J-ADNI study was approved by the ethics committee of our institution. All study subjects gave written informed consent prior to participation.

Five AD patients (three females and two males, 73.8 ± 20.7 years old) and 7 age-matched cognitively normal controls (three females and four males, 70.1 ± 9.81 years old) were assessed in this study. The patients were diagnosed with AD when they fulfilled the DSM-IV criteria for dementia and the revised National Institute of Neurologic and Communicative Disorders and Stroke-AD and Related Disorders Association criteria (Dubois et al. 2010) for probable AD and registered with the J-ADNI study as AD patients.

All of the AD patients showed positive Pittsburgh Compound B (^11^C-PIB) accumulation and all of the cognitively normal controls showed no ^11^C-PIB accumulation.

### PET/CT

^11^C-PIB-PET/CT was performed in all subjects in the Department of Nuclear Medicine of Saitama Medical University International Medical Center. Each subject received an intravenous injection of 600 MBq of *N*-methyl-[^11^C] 2-(4′-methylamino-phenyl)-6-hydroxybenzothiazole (^11^C-PIB) (Klunk et al. 2004) and underwent a 70 min list mode acquisition using PET/CT equipment with high spatial resolution (Biograph 6 Hi-Rez; Siemens Medical Solutions USA, Inc., Knoxville, TN). The combination of Fourier rebinning and ordered subsets expectation maximization with an iteration number of four, subset of 16, and an all-pass filter were used for PET image reconstruction and framing into 25 volumes: 10 sec × 6, 20 sec × 3, 60 sec × 2, 180 sec × 2, and 300 sec × 12. Attenuation correction was performed using the CT data.

Before the intravenous injection of ^11^C-PIB, high quality CT scans were obtained using the same PET/CT equipment. The scanning parameters were held constant in the helical scanning mode: 1.0-sec gantry rotation time, 130 kVp, 150–240 mAsec, 0.5:1 beam pitch, 3-mm table feed per gantry rotation, and 6 × 2 mm detector configuration. The images were reconstructed at 3-mm thickness with filtered back projection, a display field of view of 25 cm, and a reconstruction matrix size of 512 × 512.

### MRI

MRI scans were also performed in all subjects within a mean interval of 25.1 ± 8.2 days (14–40 days) before the PET/CT scan. The scans were acquired on a 1.5 T scanner using a three-dimensional (3D) sagittal magnetization-prepared rapid gradient-echo imaging sequence, which was specially adjusted for the US-ADNI protocols (http://adni.loni.ucla.edu/research/protocols/mri-protocols/). Repetition time (TR), echo time (TE), inversion time (TI), and flip angle were 9.2 msec, 40 msec, 225 msec, and 8°, respectively. The in-plane resolution was 256 × 256 (1.25 × 1.25 mm) with a slice thickness of 1.2 mm.

### Image analysis of ^11^C-PIB PET

Data analyses of ^11^C-PIB PET were performed using the PMOD software package (version 3.0; PMOD Technologies, Ltd., Zürich, Switzerland). Distribution volume ratio images referenced to the cerebellum were generated using noninvasive Logan graphical analysis (Price et al. 2005). Two experts in neuro-nuclear medicine, both with over 10 years of experience, interpreted the regional β amyloid load, focusing on whether it was consistent with a diagnosis of AD.

### Gray matter extraction from brain MRI

In statistical parametric mapping 8 (SPM8) (http://www.fil.ion.ucl.ac.uk/spm), we use the default segmentation parameters for MR images because this program is originally developed for MRI images; with very light regula-rization, warp frequency cut-off of 25 Hz, a shorter sampling distance of 3, and a customized number of Gaussians per tissue class for each patient: 2 for gray and white matter, 2 for cerebrospinal fluid, and 4 for other tissues. The MR images were then segmented to gray matter, white matter, cerebrospinal fluid, and other compartments using an unmodified version of the clustering algorithm (Ashburner and Friston 2000).

### Gray matter extraction from brain CT

**Figure 1:**
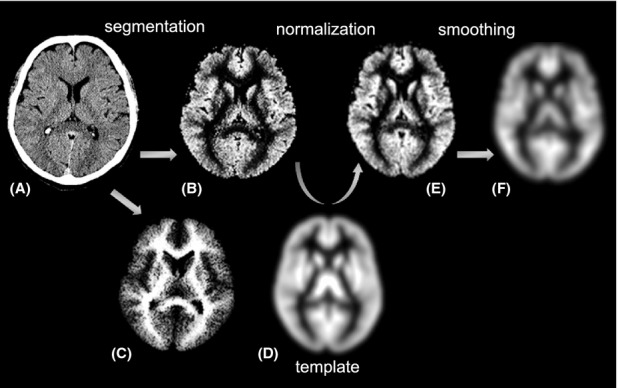
CT-based VBM procedure. (A) A slice from an original CT image. (B) Gray matter extracted from (A) using the segmentation module in SPM8. (C) White matter extracted from (B). (D) A priori template of gray matter in SPM8. (E) Spatially normalized gray matter image using (D). (F) Image smoothed to the Gaussian distribution. CT, computed tomography; VBM, voxel-based morphometry; SPM8, statistical parametric mapping 8.

We changed many default setting to the segmentation program in SPM8 taking the difference of CT and MR into account. Before using the segmentation function in SPM8, MRIcro (http://www.cabiatl.com/mricro) and Image J (http://rsb.info.nih.gov/ij) were used to preprocess the CT images. The Brain Extraction Tool (Smith 2000) in MRIcro was used to remove the head holder segment. Image J was used to make the bounding box and voxel sizes equivalent to the tissue probability maps in SPM8. In SPM8, we set the segmentation parameters with extremely heavy regularization for unbiased CT images, a larger warp frequency cut-off of 35 Hz, a shorter sampling distance of 2, and a customized number of Gaussians per tissue class for each patients: 1 or 2 for gray and white matter and 6–8 for cerebrospinal fluid and other tissues. The number of Gaussians per tissue class was adjusted for each patient until successful segmentation was achieved. The CT images were then segmented to gray matter, white matter, cerebrospinal fluid, and other compartments using an unmodified version of the clustering algorithm (Ashburner and Friston 2000) (Fig. 1).

### VBM

Segmented gray matter images, from both CT and MRI, for each original individual brain space were spatially normalized to a standard brain template (Talairach and Tournoux 1988) in a 3D space. Spatial normalization corrects for differences in brain size and shape and facilitates intersubject averaging. At the same time, voxel was modified to the same size: 2 mm × 2 mm × 2 mm. A priori gray matter images in SPM8 were used as a standard template. The gray matter images were then smoothed with a 12-mm, full-width half-maximum isotropic Gaussian kernel to use the partial volume effect to create a spectrum of gray matter intensities. The gray matter densities are equivalent to the weighted average of gray voxels located in the volume fixed by the smoothing kernel; therefore, regional intensities can be taken as being equivalent to gray matter volumes (Ashburner and Friston 2000; Ohnishi et al. 2001).

### Statistical analysis

The processed images were analyzed using SPM8, which implements the general linear model. Global gray matter in the images was treated as a nuisance confounder. Proportional scaling was used to achieve global normalization of voxel values between the images. In the analysis of patients with AD, we studied the differences in the gray matter between the cognitively normal controls versus AD patients using *t* statistics. The resulting sets of *t* values constituted statistical parametric maps: SPM (*t*), which were transformed to the unit normal distribution (SPM [*Z*]). Group analysis of gray matter volume between the AD patients and cognitively normal controls was performed using a spatial extent threshold of 123 (984 mm^3^) for CT-VBM and 381 (3048 mm^3^) for MRI-VBM contiguous voxels. Main effects used whole-brain analyses with a threshold at a voxel level of *P* < 0.005 and a cluster false discovery rate of *P* < 0.05 for the multiple comparison correction (Chumbley and Friston 2009).

## Results

**Figure 2:**
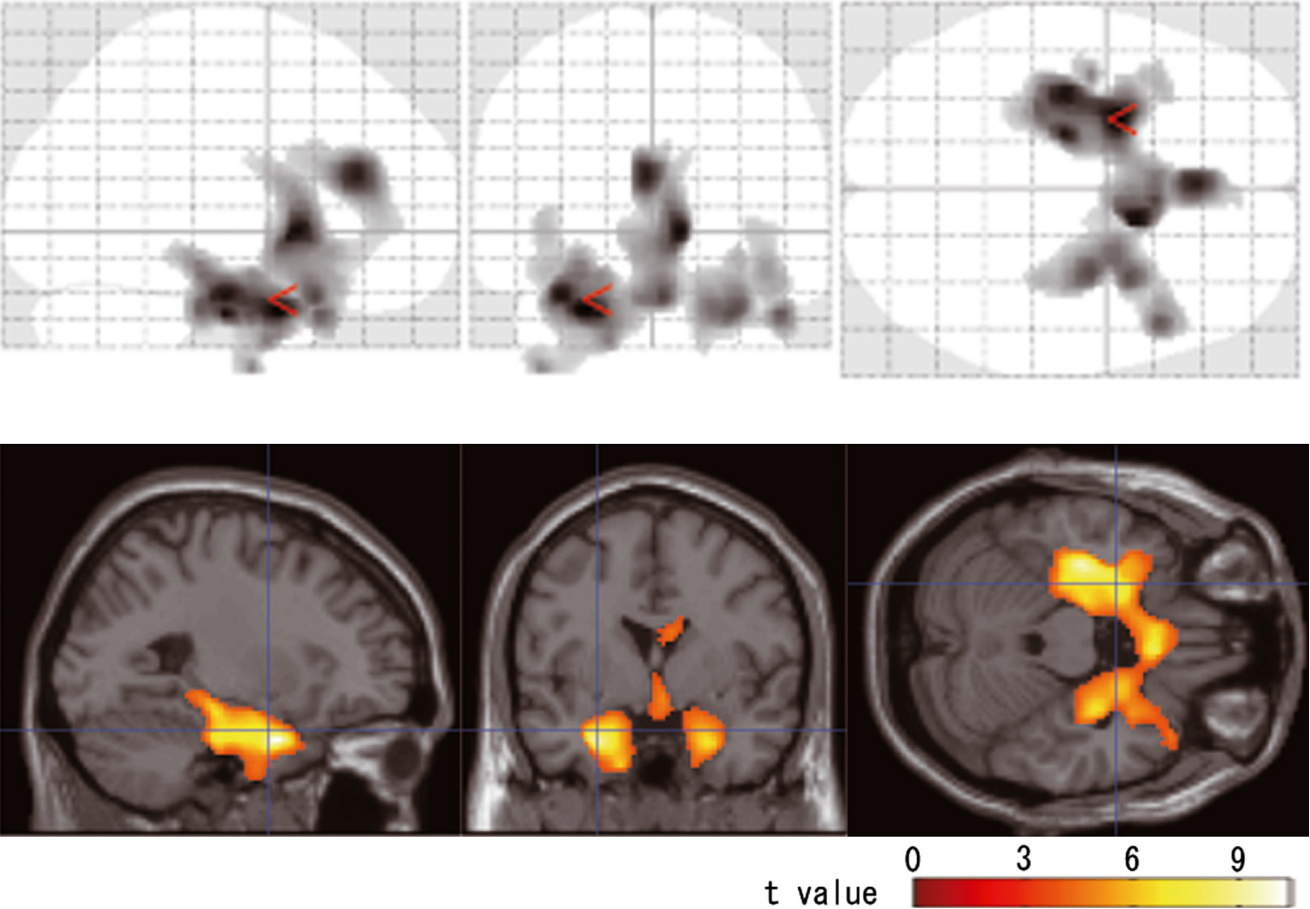
Significant reduction of regional gray matter volume is noted in the bilateral medial temporal cortex, temporopolar areas, right caudate, and anterior cingulate in AD patients with CT-VBM. Upper row: The SPM of the t statistics is displayed in a standard format as a maximum intensity projection viewed from the right hand side (left image), the back (middle image), and the top (right image) of the brain. The anatomic space corresponds to the atlas of Talairach and Tournoux. Lower row: Significance maps of decreased gray matter volume in AD patients superimposed on a T1-weighted brain MRI template image in Montreal Neurological Institute (MNI) space. The color bar represents the *t* value. AD, Alzheimer's disease; CT, computed tomography; VBM, voxel-based morphometry; SPM, statistical parametric mapping; MRI, magnetic resonance imaging.

In CT-VBM, the AD group showed a significant decrease of gray matter volume in the bilateral entorhinal cortex at Brodmann area (BA) 28, left hippocampus, in the left anterior cingulate gyri at BA 32, in the right temporopolar area, and in the right caudal head as compared to the cognitively normal group (Table 1 and Fig. 2).

**Table 1:** Results of CT-VBM

	Brodmann area	Cluster size, voxels	Peak *P* (uncorrected)	Peak *t* value	Peak *Z* value	Talairach coordinate (*x*, *y*, *z*)		
L entorhinal cortex	28	2776	0.00000216	8.955	4.595	−24	3	−24
R caudate head			0.00000351	8.483	4.493	10	12	−1
L hippocampus			0.00000435	8.279	4.447	−34	−17	−19
L anterior cingulate	32	366	0.00000668	7.885	4.354	−2	34	17
R entorhinal cortex	28	630	0.00000281	6.664	4.029	30	−7	−25
R temporopolar area	38		0.00000292	6.632	4.019	32	7	−24

Locations of gray matter volume reductions in AD patients compared with cognitively normal controls. CT-VBM, computed tomography-based voxel-based morphometry; AD, Alzheimer's disease.

**Figure 3:**
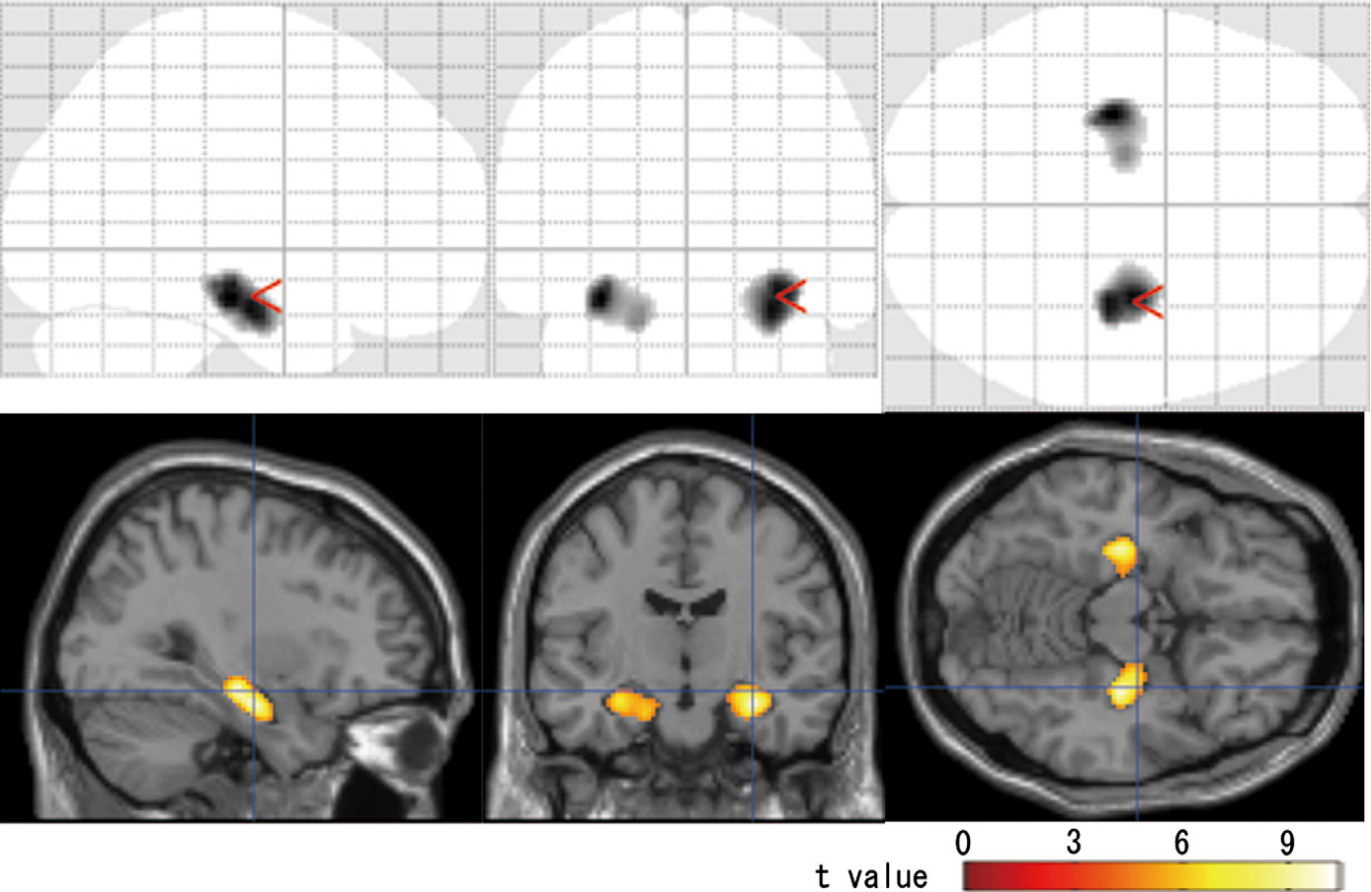
Significant reduction of regional gray matter volume is noted in the bilateral medial temporal cortex in AD patients with MR-VBM. Upper row: The SPM of the *t* statistics is displayed in a standard format as a maximum intensity projection viewed from the right hand side (left image), the back (middle image), and the top (right image) of the brain. The anatomic space corresponds to the atlas of Talairach and Tournoux. Lower row: Significance maps of decreased gray matter volume in AD patients superimposed on a T1-weighted brain MRI template image in Montreal Neurological Institute (MNI) space. The color bar represents the *t* value. AD, Alzheimer's disease; MR-VBM, magnetic resonance based voxel-based morphometry; SPM, statistical parametric mapping; MRI, magnetic resonance imaging.

In MR-VBM, the AD group showed a significant decrease of gray matter volume in the bilateral hippocampus and left entorhinal cortex at BA28 as compared with the cognitively normal group (Table 2 and Fig. 3). The most significant atrophy was observed in the left hippocampus.

**Table 2:** Results of MR-VBM

	Brodmann area	Cluster size, voxels	Peak *P* (uncorrected)	Peak *t* value	Peak *Z* value	Talairach coordinate (*x*, *y*, *z*)		
L hippocampus		381	0.00000145	9.359	4.677	−30	−18	−13
L entorhinal cortex	28		0.0000636	6.028	3.832	−16	−14	−18
R hippocampus		421	0.0000024	8.854	4.574	30	−12	−16
R hippocampus			0.0000291	8.663	4.533	34	−20	−11

Locations of gray matter volume reductions in AD patients compared with cognitively normal controls. MR-VBM, magnetic resonance-based voxel-based morphometry; AD, Alzheimer's disease.

## Discussion

In AD patients, both CT-VBM and MR-VBM demonstrated significant atrophy in the left entorhinal cortex and hippocampus and in the right entorhinal cortex or hippocampus with left-side dominancy. Our hypothesis was that, even with the gray matter segmented from CT and not from MRI, the characteristic medial temporal lobe atrophy of AD patients can be detected using the VBM procedure. This hypothesis was proved in this study.

Numerous structural MRI studies have demonstrated that atrophy of the medial temporal lobe, including the hippocampus and entorhinal cortex, is a sensitive marker of early AD (Killiany et al. 2000; Du et al. 2001). Both the entorhinal cortex and hippocampus are essential parts of the medial temporal lobe system that supports declarative memory. Neuronal loss in AD is thought to start in the entorhinal cortex and spread to other regions, such as the hippocampus (Braak and Braak 1991). Guo et al. (2010) observed gray matter volume in 13 AD patients and 14 healthy controls using MRI. In their analysis of gray matter volume, the left parahippocampal gyrus showed more significant atrophy than the right. Shi et al. (2009) found left-less-than-right asymmetry patterns by comparing hippocampal volume. These investigations seem to be in good agreement with the present results of left-side dominant atrophy.

As a result, the gray matter atrophy in AD patients observed using CT-VBM was found to be more widespread than that observed using MR-VBM. Atrophy in the right caudate head, left anterior cingulate, and right temporal pole was only observed using CT-VBM in this study. In contrast to its precise spatial and tissue resolution, MRI shows deterioration in field intensity inhomogeneity and geometric distortion depending on factors such as the machines and sequences used and the position of the brain in the coil. Because this intensity inhomogeneity affects many automatic quantitative image analyses, various algorithms for correction have been introduced (Sled et al. 1998; Arnold et al. 2001). On the contrary, brain CT has more homogeneity and much less distortion than MRI, even when using different machines or scan protocols. This increased homogeneity and reduced distortion may result in more sensitivity. There is another possibility that the segmentation parameter could contribute to the difference of CT and MR to a certain extent. This new method should be confirmed with more subjects and diagnostic accuracy should be measured.

Atrophy in the temporal pole and anterior cingulate have been reported by MRI-based VBM (Karas et al. 2003; Guo et al. 2010). These areas are also known to be pathologically involved in AD, where both amyloid deposition and neurofibrillary changes are observed (Braak and Braak 1991). The caudate head is known as a site at which amyloid and tau deposits also accumulate (Braak and Braak 1991). Madsen et al. (2010) showed a reduction of caudate volume in patients with mild cognitive impairment (MCI) and a further reduction in AD patients. They also showed a greater volume of the right caudate than the left caudate in cognitively normal controls and MCI subjects and twofold greater atrophy in the right caudate than in the left caudate in AD patients. They suggested that a possible confounding factor in some VBM studies of the caudate could result in the misregistration of anatomy across subjects along the ventricles. From our results, it was suggested that some artificial factors like cerebrospinal fluid flow may generate a confounding factor in MR-VBM; however, these are not detected on CT images.

## Conclusion

The present study modified a conventional VBM procedure using brain CT instead of MRI to detect significant atrophy in AD patients. This CT-VBM technique revealed larger areas of significant atrophy than MR-VBM. CT-VBM revealed additional significant atrophy in the anterior cingulate and right caudate head to the medial temporal areas, which were only detected in a limited manner on MR-VBM.

At the present time, though, complementary use of CT-VBM and MR-VBM is desirable, and for clinical use, a simpler and proper program for CT-VBM or an advanced scanning technique for more precise tissue contrast without heavier radiation exposure would be desirable, our results suggest that CT-VBM has the potential to replace MR-VBM for diagnosing AD.
